# The role of domestic and foreign economic uncertainties in determining the foreign exchange rates: an extended monetary approach

**DOI:** 10.1007/s12197-022-09589-5

**Published:** 2022-07-06

**Authors:** S. M. Woahid Murad

**Affiliations:** 1 School of Accounting, Economics and Finance, Curtin University, Perth, WA Australia; 2 Department of Economics, Noakhali Science and Technology University, Noakhali, Bangladesh

**Keywords:** Exchange rates, Economic policy uncertainty, Quantile regression, Panel ARDL model, Asymmetric effect, F31

## Abstract

This study extends the monetary model of the exchange rate by incorporating news-based domestic and US economic policy uncertainties (EPUs). We consider 12 developed and developing economies and use monthly data covering 2000:M1 to 2017:M2. The extended monetary model is estimated by the panel quantile regression of Machado and Santos Silva (2019) and Pesaran (2006) common correlated effects within linear and nonlinear panel ARDL frameworks. The estimates illustrate the significant effects of EPUs on developed and developing economies. The ARDL models show that the impact of EPUs is mostly a long-run phenomenon. The Wald test statistics confirm asymmetric effects of EPUs at different quantiles. Moreover, the Wald statistics also support the asymmetric effects of increasing and decreasing EPUs. Overall, domestic, and foreign economic uncertainties significantly affect developed and developing economies’ exchange rates, at least in the long run. Therefore, economic uncertainty should be considered in determining the exchange rate. This extended model might be more appropriate in the post-Covid-19 era.

## Introduction

In the aftermath of the recent global financial crisis (2007:Q4 to 2009:Q2), economists devote themselves extensively to measuring different sorts of economic uncertainty and analyzing its macroeconomic effects (for instance, Bloom 2009, 2014; Carrière-Swallow and Céspedes 2013; Jurado et al. 2015; Baker et al. 2016; Jo, and Sekkel, 2019; and Ahir et al. 2020). The COVID-19 pandemic intensifies the importance of studying the economic uncertainty further^1^. According to Hassett and Metcalf (1999), macroeconomic policies may not work properly under such uncertain prospects. For instance, Handley and Limão (2017) found that policy uncertainty adversely affects investment in export-oriented firms and technological progress, which diminishes trade and real income. Bloom et al. (2018) examined how uncertainty shaped business cycles and found that uncertainty reduced GDP by almost 2.5%. Moreover, Caggiano et al. (2017) also found that the incremental effect of unpredictable policy uncertainty on unemployment in the US economy is significantly larger during the recession. Likewise, policy uncertainty also affects the short-run and long-run money demand functions (Ivanovski and Churchill 2019; Hossain and Arwatchanakarn 2020; and Murad et al. 2021). Consequently, it is essential to encompass economic uncertainty in the conventional macroeconomic model and estimate its effect in determining macroeconomic variables. Analogously, an exchange rate determination model is not an exception.

Numerous studies examine the effect of economic risks and uncertainty on the exchange rate return and volatility. Most of these studies find a positive and statistically significant effect of economic uncertainty on the exchange rate volatility (Krol 2014; Balcilar et al. 2016; Chen et al. 2020; Bush and López Noria 2021; Abid and Rault 2021). However, such analyses do not explore the direction of movement of the exchange rate, namely, whether economic uncertainty appreciates or depreciates domestic currency. On the contrary, very few empirical works analyze the effect of economic uncertainty on the exchange rate and its expectations (Kido 2016; Beckmann and Czudaj 2017; Abid 2020) finds adverse spillover effects of US economic uncertainty on the high-yielding currencies, while the effect is positive for the Japanese currency. Like Kido (2016), Beckmann and Czudaj (2017) also find that economic, fiscal, and monetary uncertainties adversely affect Euro, Canadian dollar, and British pound sterling against the US dollar, while the Japanese yen appreciates at higher uncertainty. Furthermore, Abid (2020) revisits the determination of the exchange rate. Using a linear ARDL model, the author obtains significant short-run and long-run effects of economic uncertainty on the exchange rate. However, except for Beckmann and Czudaj (2017), most prior studies do not consider a conventional monetary approach to exchange rate determination. Therefore, most of these studies may potentially suffer from model misspecification due to omitting relevant variables. Moreover, Beckmann and Czudaj (2017) include only economic, monetary, and fiscal uncertainty indexes of the US in estimating exchange rates of Canada, Euro Area, Japan, and the UK. They do not incorporate the domestic economic uncertainties of the corresponding economies.

The novelty of this study is including both domestic and US economic policy uncertainties (EPUs) within the monetary approach to the exchange rate.^2^ Recently developed panel quantile regression with the method of moments and nonlinear panel ARDL models have been employed in the analysis to explore potential asymmetric effects of EPUs on the exchange rates.

The rest of this paper covers the following sections: Sect. 2 describes the specification of the empirical model, sources of data, and methods, while Sect. 3 discusses empirical results obtained from the analysis. Finally, the conclusion and policy recommendations are presented in Sect. 4.

## Model specification, data, and methods

Considering the purchasing power parity, money market equilibrium, and the uncovered interest parity, the monetary model of exchange rate can be expressed as

1 $${s}_{i,t}={\lambda }_{0}+{\lambda }_{1}\left({m}_{i,t}-{m}_{i,t}^{*}\right)+{\lambda }_{2}\left({y}_{i,t}-{y}_{i,t}^{*}\right)+{\lambda }_{3}\left({i}_{i,t}-{i}_{i,t}^{*}\right)+{u}_{i,t}$$

where $s$ is the exchange rate of corresponding domestic currency against the US dollar; $m$ and ${m}^{*}$ are domestic and the US money supplies; $y$ and $y$ are domestic and the US incomes; $i$ and ${i}^{*}$ are domestic, and the US interest rates, and finally, $u$ is the white noise error term. $i$ and $t$ denote $i$th economy and time, respectively. All the variables are transformed in natural log. Now, including EPUs, an extended monetary model can be obtained from Eqs. (1),

2 $${s}_{i,t}={\beta }_{0}+{\beta }_{1}\left({m}_{i,t}-{m}_{i,t}^{*}\right)+{\beta }_{2}\left({y}_{i,t}-{y}_{i,t}^{*}\right)+{\beta }_{3}\left({i}_{i,t}-{i}_{i,t}^{*}\right)+{\beta }_{4}{epu}_{i,t}+{\beta }_{5}{epu}_{i,t}^{*}+{\epsilon }_{i,t}$$

where $epu$ and ${epu}^{*}$ are domestic and the US economic policy uncertainty, respectively. These two variables are also converted in natural log. According to the prior studies, it is expected that ${\beta }_{1}=1$, implying that if the domestic money supply increases relative to the US money supply, the exchange rate will proportionately increase. Similarly, if domestic income relatively increases or the domestic interest rate relatively decreases, the domestic demand for holding money will increase. Therefore, ${\beta }_{2}$ is likely to be negative and ${\beta }_{3}$ is likely to be positive.^3^ Moreover, domestic EPU and the US EPU are expected to have negative and positive effects on the exchange rate, respectively.

This study considers five developed countries and regions, namely, Canada, Euro, Japan, Sweden, UK, and seven developing countries, i.e., Brazil, Chile, China, India, Korea, Mexico, and Russia. The period covered in the study is from 2000:M1 to 2017:M2. The countries, regions and the period of this study have been selected based on the availability of data of the considered variables. Except for EPUs, the data of all variables is collected from International Financial Statistics (IFS) published by the IMF. The domestic and the US EPUs are news-based economic uncertainty measures developed by Baker et al. (2016). They are collected from the authors’ website.^4^

Equation (2) is estimated using the panel quantile regression model of Machado and Santos Silva (2019). One advantage of this method is that it does not rely on conditional means; it is based on the method of moments. Therefore, endogenous variables can be easily accommodated in this method. To check the robustness of the findings obtained from panel quantile regression, the linear and nonlinear ARDL models are employed. Both linear and nonlinear autoregressive distributed lag (ARDL) models are estimated using Pesaran (2006). Shin et al. (2014) postulate the nonlinear ARDL model for time series analysis. However, after decomposing the policy variables in positive and negative cumulative sums, the linear ARDL model of Pesaran (2006) can be augmented in the asymmetric ARDL model (for instance, Eberhardt and Presbitero 2015; Salisu and Isah 2017). For estimating the nonlinear panel ARDL model, the domestic EPU is decomposed in the following way,

3 $$\begin{array}{l}epu_{i,t}^{pos} = \sum\limits_{j = 1}^t {\Delta epu_{i,j}^{pos}} = \sum\limits_{j = 1}^t m ax\left( {\Delta ep{u_{i,j}},0} \right)\\epu_{i,t}^{neg} = \sum\limits_{j = 1}^t {\Delta epu_{i,j}^{neg}} = \sum\limits_{j = 1}^t m in\left( {\Delta ep{u_{i,j}},0} \right)\end{array}$$

Analogously, the US EPU is decomposed as

4 $$\begin{array}{l}epu_t^{*pos} = \sum\limits_{i = 1}^t {\Delta epu_i^{*pos}} = \sum\limits_{i = 1}^t m ax\left( {\Delta epu_i^*,0} \right)\\epu_t^{*neg} = \sum\limits_{i = 1}^t {\Delta epu_i^{*neg}} = \sum\limits_{i = 1}^t m in\left( {\Delta epu_i^*,0} \right)\end{array}$$

After replacing $epu$ and ${epu}^{*}$ by their positive and negative partial sums in Eq. (2), an asymmetric ARDL model is obtained, which is.

5 $$\begin{array}{l}{s_{i,t}} = {\gamma _0} + {\gamma _1}\left( {{m_{i,t}} - m_{i,t}^*} \right) + {\gamma _2}\left( {{y_{i,t}} - y_{i,t}^*} \right) + {\gamma _3}\left( {{i_{i,t}} - i_{i,t}^*} \right) + \\{\gamma _4}epu_{i,t}^{pos} + {\gamma _5}epu_{i,t}^{neg} + {\gamma _6}epu_{i,t}^{*pos} + {\gamma _7}epu_{i,t}^{*neg} + {\nu _{i,t}}\end{array}$$

Like Eq. (2), Eq. (5) is also estimated using Pesaran (2006). The statistical significance of the asymmetric effect is justified using the Wald test. However, the panel unit root tests of all the variables are estimated before going to the panel ARDL model. Using Herwartz and Siedenburg (2008), Demetrescu and Hanck (2012), and Herwartz et al. (2019) unit root tests, it is found that all variables are integrated of order 1.^5^

## Analysis and discussion

Table 1 reports the method of moments quantile regression (MM-QR). The MM-QR shows that all macroeconomic variables of the monetary model are statistically significant, and hold expected signs for the overall economies. Income and interest rate differentials have an asymmetric effect on the exchange rates. The coefficients of these variables decline as they move to higher quantile. Like Abid (2020), it is found that domestic EPU adversely affects the exchange rate at each quantile. However, the asymmetric effect is not justified by the Wald test. Unlike domestic EPU, the US EPU has no significant impact on the exchange rates. Such outcomes may appear due to aggregation bias. After reexamining the model for developed and developing economies, it is found that domestic EPU negatively and the US EPU positively affect the exchange rates. Beckmann and Czudaj (2016) also find an instantaneous positive effect of the US EPU on the developed countries’ exchange rates.

The coefficients of both EPUs increase at higher quantiles. Hence, the asymmetric effects are significant. Notably, the impact of domestic EPU is larger than the US EPU at each quantile, i.e., the influence of domestic economic uncertainty is harsher than the foreign economic uncertainty in developed countries and regions.

In the case of developing countries, money supply and income differentials are significant and retain expected signs. However, the interest rate differentials are positive at lower quantiles and negative at higher quantiles. Higher interest rate differentials may lead to higher capital inflow in the economy (Dornbusch 1976; Frankel 1979). It may have a substantial influence on the domestic currencies of developing countries to be appreciated. Unlike developed economies, the EPUs significantly affect the exchange rate only at the lowest and highest quantiles in the developing countries. At the lowest quantiles $(\tau =0.1)$, the coefficients both domestic and the US EPUs are negative, while they are positive at the highest quantiles $(\tau =0.9)$.

The Wald test statistics show significant asymmetric effects of the EPUs on the exchange rates. In contrast to the developed countries and region, the coefficients of the US EPU exceed the coefficients of domestic EPU within the same quantiles, implying that the US economic uncertainty plays a dominant role in developing countries. Regarding the effects of economic uncertainty, Carrière-Swallow and Céspedes (2013) unveil that foreign uncertainty shocks’ spillover effect is severe on emerging economies than developed countries because of credit constraints.

To check the robustness of these findings, this study further estimates the linear and nonlinear ARDL models. According to Table 2, the extended monetary approach to exchange rates is mostly a long-run phenomenon. The money supply and income differentials are significant, and hold expected signs in the long run. The results are consistent with the MM-QR models. However, after considering nonstationary panels, the long-run coefficients of interest rate differentials of advanced economies become negative, implying that higher interest rate in advanced economies relative to the US interest rate attracts foreign investors to invest in these advanced economies and domestic investors retain themselves to invest in their own territory. Prior studies find that the interest rate differentials become an integral part of determining the international capital flow during high economic uncertainty.^6^

**Table 1:** Determination of nominal exchange rate using the method of moments-quantile regression (MM-QR)

Panel A: All Economies										Wald test for equality at different quantiles	
Variable	Quantile levels										
	0.1	0.2	0.3	0.4	0.5	0.6	0.7	0.8	0.9	0.5	0.9
$(m-{m}^{*})$	0.834^***^	0.83^***^	0.826^***^	0.824^***^	0.821^***^	0.818^***^	0.815^***^	0.81^***^	0.802^***^	2.19	2.19
	(0.011)	(0.01)	(0.01)	(0.01)	(0.01)	(0.011)	(0.012)	(0.014)	(0.018)		
$(y-{y}^{*})$	-2.619^***^	-2.461^***^	-2.32^***^	-2.214^***^	-2.12^***^	-2.017^***^	-1.886^***^	-1.716^***^	-1.41^***^	20.73^***^	20.82^***^
	(0.138)	(0.123)	(0.116)	(0.116)	(0.12)	(0.128)	(0.143)	(0.169)	(0.221)		
$(i-{i}^{*})$	0.651^***^	0.615^***^	0.583^***^	0.559^***^	0.538^***^	0.514^***^	0.484^***^	0.446^***^	0.376^***^	22.18^***^	22.28^***^
	(0.03)	(0.027)	(0.025)	(0.025)	(0.026)	(0.028)	(0.031)	(0.037)	(0.049)		
$epu$	-0.448^***^	-0.438^***^	-0.43^***^	-0.424^***^	-0.418^***^	-0.412^***^	-0.404^***^	-0.394^***^	-0.376^***^	0.47	0.47
	(0.054)	(0.048)	(0.045)	(0.046)	(0.047)	(0.05)	(0.056)	(0.066)	(0.087)		
${epu}^{*}$	-0.059	-0.058	-0.057	-0.056	-0.056	-0.055	-0.054	-0.053	-0.05	0.00	0.00
	(0.081)	(0.072)	(0.068)	(0.068)	(0.07)	(0.075)	(0.084)	(0.098)	(0.129)		
$cons.$	2.384^***^	2.781^***^	3.134^***^	3.399^***^	3.635^***^	3.895^***^	4.222^***^	4.648^***^	5.414^***^	18.38^***^	18.51^***^
	(0.368)	(0.326)	(0.308)	(0.308)	(0.319)	(0.34)	(0.38)	(0.447)	(0.588)		
Panel B: Developed Economies										Wald test for equality at different quantiles	
Variable	Quantile levels										
	0.1	0.2	0.3	0.4	0.5	0.6	0.7	0.8	0.9	0.5	0.9
$(m-{m}^{*})$	0.8^***^	0.76^***^	0.723^***^	0.668^***^	0.636^***^	0.599^***^	0.565^***^	0.53^***^	0.497^***^	113.99^***^	130.50^***^
	(0.02)	(0.018)	(0.017)	(0.016)	(0.015)	(0.016)	(0.017)	(0.018)	(0.019)		
$(y-{y}^{*})$	0.678	0.176	− 0.282	− 0.97^**^	-1.374^***^	-1.835^***^	-2.253^***^	-2.7^***^	-3.103^***^	25.33^***^	26.07^***^
	(0.564)	(0.507)	(0.471)	(0.44)	(0.434)	(0.45)	(0.477)	(0.518)	(0.564)		
$(i-{i}^{*})$	0.475^***^	0.423^***^	0.376^***^	0.305^***^	0.264^***^	0.217^***^	0.174^**^	0.128	0.087	10.25^***^	10.37^***^
	(0.092)	(0.083)	(0.076)	(0.071)	(0.071)	(0.073)	(0.078)	(0.085)	(0.092)		
$epu$	0.019	− 0.171^*^	− 0.344^***^	− 0.604^***^	− 0.756^***^	− 0.93^***^	-1.088^***^	-1.257^***^	-1.409^***^	83.91^***^	92.54^***^
	(0.111)	(0.1)	(0.095)	(0.091)	(0.087)	(0.09)	(0.095)	(0.102)	(0.11)		
${epu}^{*}$	0	0.094	0.179	0.306^***^	0.381^***^	0.467^***^	0.544^***^	0.627^***^	0.702^***^	12.99^***^	13.18^***^
	(0.148)	(0.133)	(0.122)	(0.114)	(0.113)	(0.117)	(0.125)	(0.136)	(0.148)		
$cons.$	− 0.12	0.664	1.38^***^	2.455^***^	3.086^***^	3.805^***^	4.458^***^	5.157^***^	5.786^***^	46.30^***^	48.76^***^
	(0.641)	(0.574)	(0.536)	(0.502)	(0.492)	(0.511)	(0.541)	(0.587)	(0.639)		
Panel B: Developing Economies										Wald test for equality at different quantiles	
Variable	Quantile levels										
	0.1	0.2	0.3	0.4	0.5	0.6	0.7	0.8	0.9	0.5	0.9
$(m-{m}^{*})$	0.849***	0.844***	0.841***	0.838***	0.835***	0.834***	0.832***	0.828***	0.818***	0.74	0.74
	(0.023)	(0.019)	(0.017)	(0.016)	(0.015)	(0.015)	(0.016)	(0.017)	(0.025)		
$(y-{y}^{*})$	-2.998***	-2.453***	-2.124***	-1.834***	-1.488***	-1.348***	-1.154***	− 0.735***	0.321	77.77^***^	94.94^***^
	(0.223)	(0.172)	(0.152)	(0.146)	(0.137)	(0.132)	(0.138)	(0.205)	(0.222)		
$(i-{i}^{*})$	0.562***	0.365***	0.246***	0.141***	0.016	− 0.035	− 0.105**	− 0.256***	− 0.638***	73.40^***^	89.02^***^
	(0.083)	(0.064)	(0.057)	(0.055)	(0.051)	(0.049)	(0.052)	(0.076)	(0.083)		
$epu$	− 0.188**	− 0.129*	− 0.093	− 0.062	− 0.025	− 0.01	0.011	0.056	0.17*	6.54^**^	6.65^***^
	(0.087)	(0.072)	(0.064)	(0.06)	(0.058)	(0.058)	(0.06)	(0.067)	(0.095)		
${epu}^{*}$	− 0.242*	− 0.159	− 0.108	− 0.064	− 0.011	0.011	0.04	0.104	0.266*	5.02^**^	5.08^**^
	(0.141)	(0.116)	(0.105)	(0.098)	(0.095)	(0.095)	(0.097)	(0.109)	(0.154)		
$cons.$	2.257***	2.364***	2.429***	2.486***	2.554***	2.582***	2.62***	2.703***	2.91***	0.40	0.40
	(0.649)	(0.536)	(0.483)	(0.452)	(0.437)	(0.438)	(0.447)	(0.492)	(0.709)		

Notes: (i) Figures in parentheses are standard errors. (ii) ^*^, ^**^ and ^***^ denote the significance level at 10%, 5%, and 1%, respectively, and (iii) the statistically significant Wald test results show that the estimated slope coefficients and intercepts for lower quantile $(\tau =0.1)$ are statistically different across medium $(\tau =0.5)$ and upper quantiles$(\tau =0.9)$

**Table 2:** Panel ARDL model

Linear Long-run Coefficients									Nonlinear Long-run Coefficients								
Coefficient		All		Developed			Developing		Coefficient		All		Developed				Developing
$(m-{m}^{*})$		0.660^***^		0.248^***^			0.0127^***^		$(m-{m}^{*})$		0.719^***^		0.0452^***^				-0.858^***^
		(0.0026)		(0.0025)			(0.0027)				(0.0035)		(0.0044)				(0.0034
$(y-{y}^{*})$		-1.582^***^		-1.532^***^			-1.457^***^		$(y-{y}^{*})$		-1.537^***^		-1.444^***^				-0.866^***^
		(0.0045)		(0.0072)			(0.0063)				(0.0044)		(0.0052)				(0.0064)
$(i-{i}^{*})$		0.231^***^		-0.243^***^			0.909^***^		$(i-{i}^{*})$		0.345^***^		-0.181^***^				0.666^***^
		(0.0019)		(0.0009)			(0.0027)				(0.0020)		(0.0018)				(0.0029)
$epu$		1.195^***^		0.272^***^			1.151^***^		${epu}^{pos}$		1.211^***^		0.347^***^				0.919^***^
		(0.0020)		(0.0026)			(0.0027)				(0.0020)		(0.0036)				(0.0022)
${epu}^{*}$		0.139^***^		0.198^***^			-0.236^***^		${epu}^{neg}$		1.234^***^		0.354^***^				0.826^***^
		(0.0013)		(0.0024)			(0.0017)				(0.0021)		(0.0040)				(0.0025)
									${epu}^{*pos}$		-0.104^***^		0.0511^***^				-0.297^***^
											(0.0014)		(0.0033)				(0.0015)
									${epu}^{*neg}$		-0.133^***^		0.0612^***^				-0.200^***^
											(0.0015)		(0.0035)				(0.0015)
**Linear Short-run Coefficients and the Speed of Adjustment**									**Nonlinear Short-run Coefficients and the Speed of Adjustment**								
Coefficient	All		Developed			Developing			Coefficient	All				Developed		Developing	
$ECM$	-0.0045		-0.0124^***^			-0.0049			$ECM$	-0.0046				-0.0158^**^		-0.0078	
	(0.0034)		(0.0036)			(0.0067)				(0.0031)				(0.0059)		(0.0056)	
$\varDelta (m-{m}^{*})$	0.1060		0.1510			0.0742			$\varDelta (m-{m}^{*})$	0.1070				0.1550		0.0698	
	(0.0919)		(0.1290)			(0.1370)				(0.0891)				(0.1250)		(0.1320)	
$\varDelta (y-{y}^{*})$	-0.0073		0.0158^**^			-0.0201			$\varDelta (y-{y}^{*})$	-0.0072				0.0180^***^		-0.0194	
	(0.0092)		(0.0058)			(0.0137)				(0.0091)				(0.0044)		(0.0139)	
$\varDelta (i-{i}^{*})$	0.0232		-0.0131			0.0531^*^			$\varDelta (i-{i}^{*})$	0.0241				-0.0146		0.0520^*^	
	(0.0185)		(0.0187)			(0.0240)				(0.0181)				(0.0160)		(0.0248)	
$\varDelta epu$	0.0161		0.0329			0.0050			${\varDelta epu}^{pos}$	0.0928				0.2050		0.0080^***^	
	(0.0123)		(0.0303)			(0.0026)				(0.0864)				(0.2030)		(0.0024)	
$\varDelta {epu}^{*}$	0.0073^**^		0.0009			0.0122^***^			${\varDelta epu}^{neg}$	-0.0640				-0.1540		0.0006	
	(0.0024)		(0.0031)			(0.0028)				(0.0650)				(0.1540)		(0.0032)	
									${\varDelta epu}^{*pos}$	0.0111^***^				0.0060		0.0154^***^	
										(0.0033)				(0.0056)		(0.0043)	
									${\varDelta epu}^{*neg}$	0.0021				-0.00630^*^		0.00779^*^	
										(0.0030)				(0.0028)		(0.0035)	
Test of asymmetric effects					All				Developed			Developing					
					$epu$			${epu}^{*}$	$epu$		${epu}^{*}$	$epu$			${epu}^{*}$		
${\omega }^{LR}$					6889^***^			22,971^***^	192^***^		873^***^	47,875^***^			26,273^***^		
${\omega }^{SR}$					1.07			4.65^**^	1.01		2.76^*^	8.06^***^			1.93		

Notes: ^***^, ^**^ and ^*^ indicate 1%, 5% and 10% levels of significance, respectively. The parentheses values show the standard errors. Westerlund et al. (2019) proposed a fixed-T variance estimator that has been used for pooled coefficients in Pesaran (2006). This estimator corrects the heteroscedasticity problem and considers fixed time variance for panels

Furthermore, the linear ARDL model shows that domestic and the US EPUs depreciate domestic currencies of advanced economies in the long run. The nonlinear ARDL model also estimates positive coefficients positive and negative cumulative sums of EPUs, implying that the exchange rate always increases in the long run when domestic and the US economic uncertainties increase or decrease. Although these uncertainty coefficients hold the same sign, their magnitudes are statistically different according to the Wald test. Like the quantile regression, the ARDL models also show that the effect of domestic EPU is larger than the US EPU in the advanced economies. However, domestic EPU has no significant impact on the exchange rates in developed economies during the short run. In the short run, only the US EPU appreciates domestic currencies when the US EPU decreases. The Wald test reports that only the US EPU has an asymmetric effect on the exchange rate in the short run.

On the contrary to the advanced economies, the linear and nonlinear panel ARDL models for developing countries show that domestic EPU positively and the US EPU negatively affect the exchange rates in the long run. The Wald test justifies the asymmetric long-run effects of domestic and foreign EPUs on the exchange rate. In the short run, domestic EPU depreciates the domestic currencies of developing countries when the EPU increases. The foreign EPU positively impacts the exchange no matter whether the US EPU increases or decreases in the short run. However, the Wald test only supports the short-run asymmetric effect of domestic EPU. The adjustment parameters hold the expected sign and are also statistically significant only for advanced economies, and the value is higher in the nonlinear ARDL model.

## Conclusions

This study attempts to extend the monetary model to exchange rates by encompassing the domestic and the US economic uncertainties. Relying on the monthly data of advanced and developing economies over 2000:M1 to 2017:M2, it is found that both domestic and foreign economic uncertainties significantly affect the equilibrium exchange rates, at least in the long run. In the case of advanced economies, the effect of domestic EPU exceeds the foreign EPU. In contrast, the foreign EPU is more influential in developing countries. Therefore, economic uncertainty may be considered as a ‘scapegoat’ in the model of Bacchetta and van Wincoop (2004, 2013). After considering the effects of economic uncertainty in the monetary model, all the macroeconomic variables are statistically significant, and hold expected signs in most cases. It supports Engel et al. (2008) findings that the monetary model still has explanatory power in determining exchange rates. According to the findings of this study, economic uncertainty emerges as an integral part of exchange rate determination. The notion would be more relevant in the post-Covid-19 era.

However, Caggiano et al. (2014) and Kido (2016) find the spillover effects of uncertainty shocks severe during the recessionary period. Therefore, this extended monetary model can be reinvestigated after considering economic recessions.

## Footnotes

1 A comprehensive literature survey on COVID-19 and economic uncertainty can be found in Baker et al. (2020).

2 Bilson (1978) and Frankel (1979) are among the classic articles on the monetary approach to exchange rates. For a comprehensive literature survey including recent studies, see Xie and Chen (2019).

3 These hypotheses are derived from Bilson (1978). However, Dornbusch (1976) and Frankel (1979) argue that if interest rates differential rises, capital inflow will also increase in the domestic economy. Therefore, the interest rate differential and exchange rate are expected to move in opposite direction, i.e., ${\beta }_{3}$ is supposed to be negative.

4 For detail about the construction of EPU index and the database see www.policyuncertainty.com.

5 To save the space, the results of unit root test are not presented in the paper. However, the results are available from the author upon request.

6 However, numerous studies postulate that the interest rate differentials do not determine the international capital flow (Grubel 1968; Haynes 1988).
